# Credit to Whom Credit Is Due

**Published:** 1855-01

**Authors:** 


### EDITORIAL.

*Credit to whom Credit is due.*

"Render to Cæsar the things that are Cæsar's," was a reply that puzzled the wise men of Judea not a little; and it was brought forcibly to my mind while reading the little volume entitled, Bernard and Robin on the Blood, by Dr. Walter F. Atlee, which was noticed in the December number of the Journal by my editorial colleague, Dr. Johnson. Whether it is the fault of M. Bernard or of his reporter, I do not know, but it is certain that one not well versed in medical literature would get the impression, from reading this book, that very little had been done in the way of investigating the composition of the blood and it changes, outside of Paris. Some facts are stated as though they had only recently been brought to light by M. Bernard, which have been known to the profession for many years; and both the distinguished French savan and his reporter, Atlee seem to be entirely ignorant of some things which have been done on this side of the Atlantic. For instance in speaking of the blood in the renal vein, compared with that in the arteries, the book before us says: "this blood," (from the renal vein) "as Simon has shown, and he alone has made an analysis, comparing it with the arterial, contains no fibrine. ** * * There is less water in the vein, for the urine carries it off, and the solid matters are in a greater proportion, because there is less water. If the fibrine be added to the albumen in the arterial blood, it equals the albumen in the venous, as if the fibrine had been transformed into it." And again: "The albumen *augments in the renal* venous blood, and M. Bernard is disposed to believe that it is owing to a transformation of the fibrine; for looking at the ciphers in the tables, we see that it is just what is wanted to compensate for the difference in the albumen."

Now, these brief extracts contain no less than *three* important errors viz: 1st, Simon is not the *only* man who has made an analysis of renal venous blood. 2nd, It is not true that in all instances renal venous blood contains *no* fibrine. 3d, The amount of Albumen is not always *positively augmented*; nor augmented in strict proportion to the loss of fibrine. If Dr. Atlee will turn to a paper read by me before the American Medical Association, at Charleston in May, 1851, entitled "An Experimental Inquiry concerning some points in the Vital Processes of Assimilation and Nutrition;" he will find among other things, the following quotation, viz:

"There have been numerous analyses of venous blood in comparison with arterial, but without any reference to the particular parts from which the venous blood had been returned. The one exception to which allusion was just made, was by Simon, who procured the blood from the renal veins, the hepatic veins, and the aorta of a horse, and subjected each specimen to careful analysis, particularly in reference to the quantity of *fibrine* contained. The results are given on page 139 of his work on the Chemistry of Man, and are as follows, viz.:

| | Renal Vein. | Hepatic Vein. | Aorta. |
|---|---|---|---|
| Water, | 778,000 | 725,000 | 790,000 |
| Albumen, | 99,230 | 130,000 | 90,300 |
| Fibrine, | 000 | 2,500 | 8,200 |
| Whole solid matter, | 222,000 | 275,000 | 210,00 |

This presents truly a striking result; the blood returning from two of the largest secreting organs in the body is found to contain far less *fibrine* and *water*, and more *albumen* than that from the aorta. But there are two circumstances which render this analysis unsatisfactory. First, the horse from which the blood was taken, was not healthy, and was in a *starved* condition. Second, the quantity of blood obtained by Simon from the renal veins, was insufficient to determine accurately the proportion of fibrine, being only 50 grs. To obviate these objections, and at the same time add another important element to the comparison, I procured a large, healthy and active dog, and procured some blood from the renal vein, the illiac vein, and the illiac artery, in the following manner. The dog was stunned by a blow on his head, the abdomen quickly laid open, and a ligature passed around the renal vein near its entrance into the ascending cava, when on puncturing the vein 590 grains of blood flowed readily into a clean cupping glass. A ligature was next passed around the illiac vein, and on puncturing it, 771 grains of blood were collected in another cup. The illiac artery, being now easy of access, was punctured and the blood in a full pulsating stream, and received into a third cup, to the amount of 1816 grains. * * * * All these specimens of blood were most carefully analyzed, following the method recommended and so long practised by Andral and Gavarret, with the exception of the mode of separating the fibrine. For accomplishing this most accurately, I fully agree with Mr. Bence Jones, preferring to allow the blood to coagulate perfectly, and then enclose the whole clot in a clean, fine linen cloth, and wash it with distilled water until the red corpuscles are entirely removed. The results in tabular form are as follows, viz:

| | Blood from the Iliac Artery in 1000 parts. | Blood from the Iliac Vein in 1000 parts. | Blood from the Renal Vein in 1000 parts. |
|---|---|---|---|
| Water, | 812 20 | 811,40 | 802,40 |
| Solid Matter, | 187,80 | 188.60 | 196,60 |
| Of which was fibrine, | 2,20 | 2,50 | 1,70 |
| Extractive Matter and Albumen, | 98 10 | 89.50 | 98 50 |
| Red Corpuscles. | 82.50 | 92.70 | 92,20 |
| Salts, | 5,00 | 3,90 | 4,20 |

From this extract, which together with the whole paper, was published in the North Western Medical and Surgical *Journal* for September, 1851, it will be seen that the blood of the renal vein *was analyzed* by me *five* years since; that the blood so analyzed *did* contain *fibrine*, though in a much smaller proportion than the arterial blood of the same animal; and finally that the albumen in the blood of the renal vein, instead of being increased in proportion to the diminution of fibrine, is not positively increased at all.

Its slight *apparent* increase being less than it should be from the diminished proportion of water. It is not at all strange that M.

Bernard, should be ignorant of the paper containing the foregoing analyzes; but the *representatives* of our profession abroad should be well informed in regard to the state of medical investigations at home: and especially should this be the case with such as take it upon themselves to publish books after they return home. The largest portion of the paper read by me to the American Medical Association in 1851, was occupied with the results of a somewhat extensive series of experiments in relation to the influence of diet and drinks on the functions of respiration and calorification. Some of these results I find are not in consonance with the views of M. Bernard.

For instance he alledges that the temperature of the body is the highest at the *end* of the active period of digestion, that is, about six hours after taking food. I made numerous experiments in reference to this point, and have always found the highest temperature *during* the most active period of digestion, instead of at the end of that process. In the paper alluded to, the results of some of these experiments are given as follows, viz: "The observations were repeated six times each day, at 7½ o'clock A. M., half an hour before taking food; at 10½ A. M., two and a half hours after breakfast; at 12½ P.M., immediately before dinner; at 3½ P.M., two and a half hours after dinner; at 5½ P.M., half an hour before tea; and at 8 P.M., two hours after tea. The following is the average result of 16 days' observations under the influence of an ordinary mixed diet, viz:

| | 7½ o'clock, A.M. | 10½ A.M. | 12½ P.M. | 3½ P.M. | 5½ P.M. | 8 P.M. |
|---|---|---|---|---|---|---|
| Av. Temperature. | 94° F. | 96° F. | 95° 2 F. | 96° 7 F | 95° 2 F | 96° F. |
| Highest. | 94° 5 F. | 97°3 F | 96° F. | 97 2 F. | 95° 5 F | 96° F. |
| Lowest, | 93° 7 F. | 96°6 F. | 94° F. | 96° 3 F. | 95° F | 96° F. |

From this table the inference is plain that the temperature of the body is uniformly from one to two degrees higher during the active stage of digestion, that is, about *two* hours after eating, than after the digestive process is fully completed.

Similar experiments were made in reference to the temperature of the system under the influence of a diet exclusively corbonaceous, and under that of a diet wholly nitrogenous; the results of which are given in the paper, and correspond closely with the above so far as relates to the influence of digestion. I am aware that the paper to which I have alluded as having been read to the American Medical Association in 1851, attracted very little attention, and after its publication, I do not know that it was either copied or commented on by any of the medical journals in the country. And though the experiments, observations and analyses, detailed in it, cost me much tedious labor and time, yet I might have persuaded myself that the results were really of no importance, had it not been that similar experiments and observations have been made and published in European journals from time to time since, and these have invariably been copied and sent the round of American journals as though they possessed much interest.

This fact seemed to justify me in again calling attention to my own work. It has been frequently alleged as a defect in American Medical Literature, that it contained so little matter of original experimental character.

And probably the best mode of supplying the defect, would be to render full and prompt credit to such individuals as do attempt investigations of this character; for there are few things more discouraging, than to spend much time and labor on experimental inquiries and see the results entirely neglected or overlooked until some European savan takes up the same subject, perhaps spends less than half the time, and announces results very similar, which are caught up with avidity and copied into every journal in the Union. D.

